# Supplementary material for: Causal Relationship between Aging and Anorexia Nervosa: A White-Matter-Microstructure-Mediated Mendelian Randomization Analysis
Source: Biomedicines. 2024 Aug 16;12(8):1874. doi: 10.3390/biomedicines12081874 (PMC11351342; doi:10.3390/biomedicines12081874)
Supplement: Supplementary file 1 [file biomedicines-12-01874-s001.zip › Supplementary Figure/Figure S8/Leaveoneout plot of MR analysis for TL to ubm-b-1543.pdf]

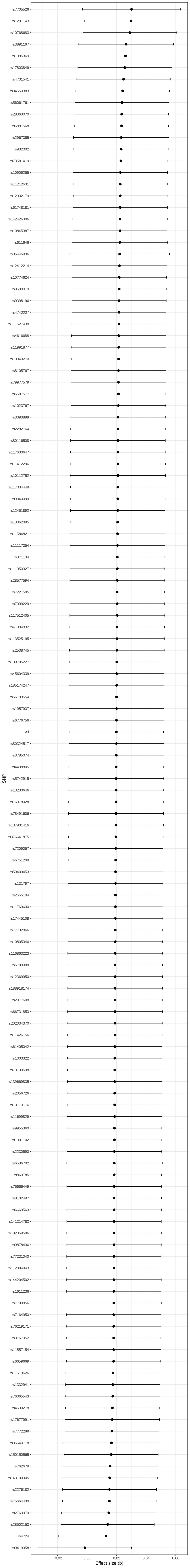
rs7705526
rs1291143
rs10768683
rs3891167
rs1985369
rs17803849
rs4731541
rs34550383
rs56061761
rs28363070
rs6881568
rs2967355
rs932002
rs73581419
rs10905255
rs11212631
rs12932179
rs61748181
rs142426306
rs10845387
rs611646
rs35446936
rs12412214
rs10774624
rs9600019
rs9398196
rs4743037
rs111527438
rs4616688
rs11991877
rs10840270
rs8105767
rs79977579
rs6587577
rs1023767
rs3093888
rs2282764
rs80116508
rs117630647
rs11412296
rs10112752
rs117034449
rs9940099
rs12451892
rs13062095
rs11584821
rs11117354
rs871134
rs111950327
rs28577594
rs7221585
rs7099229
rs117512405
rs41304832
rs113525195
rs2538745
rs139795227
rs45604339
rs185174247
rs56799554
rs1957937
rs6776756
All
rs80324517
rs3785074
rs4498805
rs5742915
rs13230646
rs16978028
rs78491606
rs137901416
rs376641875
rs7209057
rs6751209
rs59409453
rs131797
rs2555104
rs11769630
rs17445108
rs77732866
rs10805346
rs116863223
rs6790988
rs12369950
rs188918174
rs2977608
rs66731853
rs202034370
rs11426156
rs61405042
rs1003322
rs73730598
rs139669835
rs2056726
rs10773176
rs11699829
rs9955360
rs1907702
rs2230590
rs6536702
rs869785
rs76666449
rs8102497
rs6669563
rs141214782
rs182059586
rs9878436
rs77231040
rs112394943
rs144204502
rs1611236
rs7790856
rs7164950
rs76219171
rs3767952
rs11557154
rs6659669
rs11579626
rs1332941
rs76065543
rs4530278
rs17677991
rs7772289
rs35640778
rs150150565
rs762679
rs143190905
rs2276182
rs75664430
rs2763979
rs28502153
rs4724
rs9419958
SNP
-0.02
0.00
0.02
0.04
0.06
Effect size (b)
